# Case of Removal of Half of the Lower Jaw

**Published:** 1860-01

**Authors:** J. Mason Warren


### CASE OF REMOVAL OF HALF OF THE LOWER JAW.

Read before the Boston Society for Medical Improvement, and communicated for the Boston Medical and Surgical Journal.

BY J. MASON WARREN, M D.

James W., 56 years of age, applied to me in the early part of September, 1859, for a tumor about the size of a hen's egg, occupying the angle and horizontal part of the right side of the lower jaw. The disease had commenced twelve years previously, with a numbness in the jaw, followed by swelling; it went on gradually for two or three years, during which the three posterior teeth were removed. About three years since, the pain became excessive, when an opening was made into it with a lancet, and a discharge of fluid took place, attended with considerable relief.

When he presented himself, the outlines of the jaw had disappeared, and the place was occupied by a smooth, round, shell-like tumor, which extended from the canine tooth backward, rising a little upon the ramus of the jaw. The tumor projected inward, pressing upon the tongue, lifting up the palate, and obstructing about one-third of the aperture of the posterior fauces. The health of the patient was pretty good, and the principal inconvenience given to him by the tumor arose from the obstruction to deglutition, and the affection of the voice; it was also the seat of more or less uneasiness. The disease thus far did not seem to have broken out, and invaded the soft parts.

There appeared therefore, to be no question as to the propriety of its removal by operation; the only doubt was, whether to remove the jaw at the articulation, or saw off the bone just below. As it was doubtful, however, whether the disease might not extend out of sight nearly up to the articulation, as it appeared to do on looking at it from within the mouth, added to the advice given by some distinguished surgeons, that it is always better to remove the bone at the articulation, on account of the remaining portion being dragged forward by the temporal and pterygoid muscles, thus causing much irritation in the flesh, I decided on the following method of procedure, although I should not put much weight in the latter objection, not having found it to hold good in practice, and there being always more or less danger, in disarticulating the bone, of dividing the facial nerve and producing paralysis of the face. I was therefore principally governed by the apparent extent of the disease.

The patient being fully etherized, an incision was made through the skin, commencing just in front of the articulation and half an inch below the zygoma, which was carried in a semi-circular sweep backward round over the tumor, skimming lightly over the facial artery, and terminating upon the chin, about an inch and a half from the lip, opposite the second incisor tooth. The flap was now partially dissected up, and the facial artery cut and tied, in the way I had proposed before the operation. The dissection was then commenced more freely, when the facial artery, which had been lifted near its origin by the tumor, was cut again, although the incisions were not carried any further below than previously. The blood at once spouted out with such force as to completely fill my eyes, and obscure the operation. I mention this fact to show, how the best concerted plan may be frustrated by the anatomical displacement of the parts induced by the growth of tumors in their neighborhood. Although the flow of blood was arrested at once, the patient became quite faint, and was obliged to be placed in the horizontal position for a few moments, which is also worth adverting to, as so rarely occurring in the course of surgical operations where the patient is kept up by the stimulus of the ether, and which probably, previous to its introduction, as often occurred from the exhaustion of the system by pain as from the loss of blood. A tooth being removed, the jaw was partially sawn through with a small saw, and the section completed by the cutting forceps. Hitherto no blood had entered the patient's mouth, notwithstanding the profound state of etherization. The tumor was now dissected away from the tongue and soft palate, and being seized by the left hand was depressed, so as to allow the attachment of the temporal muscle to the coranoid process to be cut away with the scissors. The disarticulation was completed by cutting into the articulation from the inside with a knife, care being taken to keep close to the bone and not wound the internal maxillary artery. The only vessel of any size requiring ligature was the inferior maxillary.

The wound was allowed to drain for two or three hours, when it was closed by six or eight points of suture. The patient had scarcely a bad symptom, and the wound was almost entirely healed at the end of a couple of weeks, when he left town.

On making a section of the tumor with the saw, the jaw was found expanded into a shell, the contents being a soft grey matter.

It may be worth mentioning, that in depressing the jaw for disarticulation, although done with great care, the ramus partly gave way in the tumor, against which occurrence, a caution is given in some work on surgery. The facial nerve, and so far as could be ascertained, the parotid duct, seemed to have escaped the incision, the dissection for the disarticulation of the bone being made, after the tumor was sufficiently free, as far as possible from the inside.

